# Glucocorticoid Receptor Antagonism Upregulates Somatostatin Receptor Subtype 2 Expression in ACTH-Producing Neuroendocrine Tumors: New Insight Based on the Selective Glucocorticoid Receptor Modulator Relacorilant

**DOI:** 10.3389/fendo.2021.793262

**Published:** 2022-01-04

**Authors:** Rosario Pivonello, Pamela N. Munster, Massimo Terzolo, Rosario Ferrigno, Chiara Simeoli, Soraya Puglisi, Utsav Bali, Andreas G. Moraitis

**Affiliations:** ^1^ Dipartimento di Medicina Clinica e Chirurgia, Sezione di Endocrinologia, Università Federico Il di Napoli, Naples, Italy; ^2^ Department of Medicine (Hematology/Oncology), University of California San Francisco, San Francisco, CA, United States; ^3^ Department of Clinical and Biological Sciences, San Luigi Gonzaga Hospital, University of Turin, Orbassano, Italy; ^4^ Bioscience Department, Sygnature Discovery Ltd, Nottingham, United Kingdom; ^5^ Drug Research and Development, Corcept Therapeutics, Menlo Park, CA, United States

**Keywords:** glucocorticoid, cortisol, somatostatin, relacorilant, neuroendocrine tumor, adrenocorticotropic hormone, Cushing disease, ectopic ACTH syndrome

## Abstract

Somatostatin exhibits an inhibitory effect on pituitary hormone secretion, including inhibition of growth hormone and adrenocorticotropic hormone (ACTH), and it can have antisecretory and antitumor effects on neuroendocrine tumors (NETs) that express somatostatin receptors. Although the precise mechanism remains unclear, the finding that glucocorticoids downregulate somatostatin receptor subtype 2 (SSTR2) expression has been used to explain the lack of efficacy of traditional SSTR2-targeting analogs in patients with ACTH-secreting NETs. Glucocorticoid receptor (GR) antagonism with mifepristone has been shown to reverse the glucocorticoid-induced downregulation of SSTR2; however, the effects of GR modulation on SSTR2 expression in ACTH-secreting NETs, particularly corticotroph pituitary tumors, are not well known. The current study presents new insight from *in vitro* data using the highly selective GR modulator relacorilant, showing that GR modulation can overcome dexamethasone-induced suppression of SSTR2 in the murine At-T20 cell line. Additional data presented from clinical case observations in patients with ACTH-secreting NETs suggest that upregulation of SSTR2 *via* GR modulation may re-sensitize tumors to endogenous somatostatin and/or somatostatin analogs. Clinical, laboratory, and imaging findings from 4 patients [2 ACTH-secreting bronchial tumors and 2 ACTH-secreting pituitary tumors (Cushing disease)] who were treated with relacorilant as part of two clinical studies (NCT02804750 and NCT02762981) are described. In the patients with ectopic ACTH secretion, SSTR2-based imaging (Octreoscan and ^68^Ga-DOTATATE positron emission tomography) performed before and after treatment with relacorilant showed increased radiotracer uptake by the tumor following treatment with relacorilant without change in tumor size at computed tomography. In the patients with Cushing disease who received relacorilant prior to scheduled pituitary surgery, magnetic resonance imaging after a 3-month course of relacorilant showed a reduction in tumor size. Based on these findings, we propose that GR modulation in patients with ACTH-secreting NETs upregulates previously suppressed SSTR2s, resulting in tumor-specific antisecretory and anti-proliferative effects. The effect of relacorilant on pituitary corticotroph tumors is being investigated in an ongoing phase 3 study (NCT03697109; EudraCT 2018-003096-35).

## Introduction

Somatostatin receptors (SSTRs) are expressed in organs and tissues throughout the body (1–3) as well as in many different tumor types, including neuroendocrine tumors (NETs) (4–7)—a heterogeneous group of neoplasms (eg, pituitary tumors, carcinoid tumors, gastroenteropancreatic tumors, phaeochromocytomas, medullary thyroid carcinomas, and small cell tumors of the lung and prostate) arising from neuroendocrine cells. SSTRs include five different subtypes (SSTR1-5) belonging to the G-protein-coupled receptor class (1, 7). SSTR2 and SSTR5 are predominately expressed in endocrine tissues (eg, pituitary gland), and SSTR2 is one of the most abundantly expressed receptor subtypes in many NETs (2, 5, 6, 8). The presence of SSTR2 on NETs has led to the use of synthetic somatostatin analogs (eg, octreotide and lanreotide) (9–13) and radiolabeled somatostatin analogs (eg, ^111^In-pentetreotide [Octreoscan] and ^177^Lu-DOTATATE) (14, 15) for tumor localization and treatment.

Somatostatin is a potent inhibitor of pituitary hormone secretion, including inhibition of growth hormone and adrenocorticotropic hormone (ACTH) secretion (1, 8, 16). Similarly, somatostatin analogs have antisecretory effects and show antitumoral activity (9–12). However, many patients with NETs develop resistance or do not respond to somatostatin analogs targeting SSTR2. For instance, octreotide is partially effective in patients with extra-pituitary corticotroph tumors responsible for ectopic Cushing syndrome or ectopic ACTH secretion and is generally ineffective in patients with pituitary corticotroph tumors responsible for the most common form of ACTH-dependent Cushing syndrome, namely, Cushing disease (17, 18). Pituitary corticotroph tumors have lower expression of SSTR2 and higher expression of SSTR5 and dopamine type 2 (D2) receptors (19, 20), leading to the clinical use of dopamine agonist cabergoline, which has high affinity for D2, and the multi-somatostatin analog pasireotide, which has high affinity for SSTR5 (21, 22), over analogs targeting SSTR2.

The lack of efficacy of somatostatin analogs targeting SSTR2 in patients with Cushing disease supports the hypothesis that SSTR2 is downregulated by glucocorticoids. *In vitro* studies have shown that dexamethasone treatment of At-T20 corticotroph tumor cells induced significant suppression of SSTR2 messenger ribonucleic acid (mRNA) expression (23). Glucocorticoids have also been shown to attenuate the inhibitory effects of octreotide on ACTH release *in vitro* (18, 23). This downregulation by glucocorticoids might explain not only the lack of efficacy of SSTR2-targeting somatostatin analogs in patients with Cushing disease but also their partial effect in patients with ectopic ACTH syndrome. The ACTH-secretory capacity of these latter patients’ tumors has been shown to be more “resistant” to the negative feedback of cortisol excess, as an intact glucocorticoid signaling pathway is not always present within these cells (24–27).

The glucocorticoid receptor (GR) antagonist mifepristone, a non-selective steroidal GR antagonist with progesterone receptor activity, has been shown to reverse the inhibitory effects of glucocorticoids on SSTR2 mRNA expression in the human neuroendocrine cell lines BON (carcinoid) and TT (medullary thyroid carcinoma) (27). Treatment with dexamethasone resulted in 71% and 69% reductions in SSTR2 mRNA expression in BON and TT cells, respectively. Co-administration with mifepristone completely inhibited the dexamethasone-mediated downregulation of SSTR2 mRNA. The *in vitro* effects of mifepristone on SSTR2 expression in corticotroph tumor cell lines have not been studied. A clinical report of two patients with ectopic ACTH syndrome treated with mifepristone noted upregulation of SSTR2 on diagnostic imaging (24). However, mifepristone treatment in patients with ACTH-dependent Cushing syndrome, including ectopic ACTH syndrome, has not been shown to affect tumor growth or decrease the ACTH-secretory capacity of the tumor (28, 29).

Relacorilant (CORT125134, Corcept Therapeutics, Menlo Park, CA) is a highly selective non-steroidal GR modulator (SGRM) that modulates cortisol activity (30) and, unlike mifepristone, lacks progesterone receptor activity. Relacorilant is under clinical investigation for the treatment of patients with endogenous Cushing syndrome (NCT03697109, NCT04308590, and NCT04373265). The effects of relacorilant on SSTR2 expression have not been previously assessed, and the diagnostic and therapeutic implications of the relationship between GR modulation and SSTR2 in patients with ACTH-secreting NETs remain unclear.

## Hypothesis

**Figure 1:**
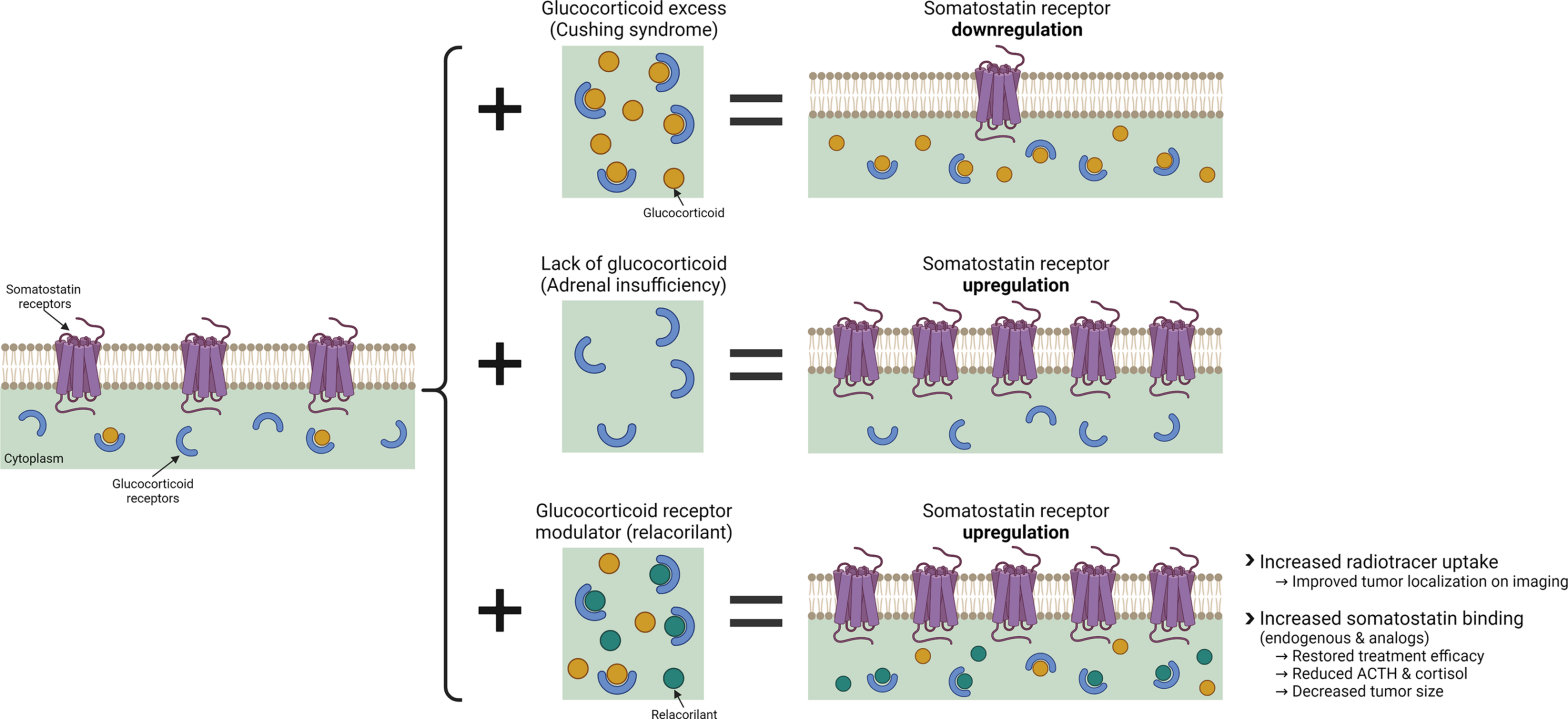
The effects of glucocorticoid and glucocorticoid modulation with relacorilant on SSTR regulation. Created with BioRender.com.

This report provides new insight into the relationship between GR modulation and SSTR2, suggesting that GR modulation with relacorilant may overcome the glucocorticoid-induced suppression of SSTR2, enabling SSTR2-mediated effects in ACTH-secreting NETs (**Figure 1**). Presented in this report are *in vitro* laboratory data and findings from several clinical observations supporting the hypothesis that SGRM-induced upregulation of SSTR2 by relacorilant may enhance tumor localization *via* SSTR imaging and may also sensitize tumors to the antitumor effects of somatostatin and its analogs.

## Experimental Study: Effect of Relacorilant on SSTR2A/2B mRNA Expression

An *in vitro* analysis was undertaken to assess, for the first time, the effects of relacorilant on SSTR2 mRNA in a mouse pituitary corticotroph cell line. In mice, two isoforms of SSTR2, SSTR2A and SSTR2B, have been identified, with human tissues expressing SSTR2A almost exclusively (31, 32). Using the murine At-T20 cell line, a well-studied corticotroph model (23), an assessment of the effects of dexamethasone alone on SSTR2A/2B mRNA levels was conducted followed by an assessment of the effects of dexamethasone and relacorilant combined.

At-T20 cells were cultured in complete medium, and mRNA expression levels of SSTR2A and SSTR2B were determined upon pretreatment for 24 hours across a dexamethasone concentration gradient (detailed Methods can be found at the end of the report). Dexamethasone treatment (100 nM) resulted in a 3-fold and 2.4-fold reduction in SSTR2A and SSTR2B mRNA levels, respectively.

**Figure 2:**
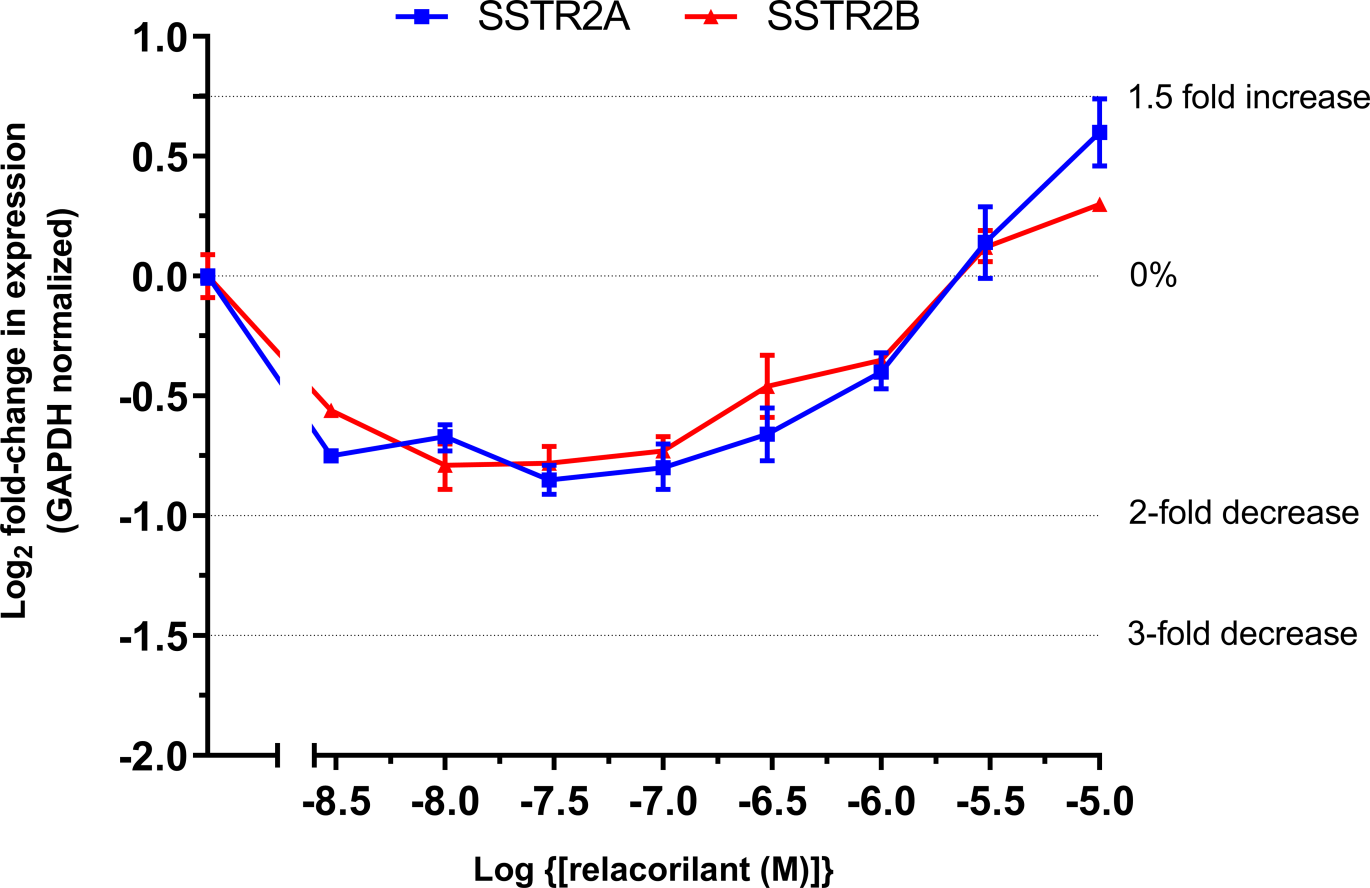
Log_2_ fold change in SSTR2 mRNA in murine At-T20 cells upon treatment with increasing concentrations of relacorilant for 24 h in the presence of 100 nM dexamethasone. 0%, 2-fold, and 3-fold inhibition and 1.5-fold increase in levels are highlighted by dotted lines on the y-axis. Zero relative expression is in the absence of dexamethasone. Data points show average fold change compared to baseline and SD error bars. Data are technical replicates with an underlying n=1.

Relacorilant inhibited the dexamethasone-mediated reduction of SSTR2A/2B mRNA in a concentration-dependent manner (**Figure 2**). At relacorilant concentrations greater than 1 µM, an increase in SSTR2A/2B mRNA levels above basal (untreated) levels was observed, reaching an ~1.5-fold increase at 10 µM, the highest relacorilant concentration tested.

## Clinical Observations

Four patients with NETs received investigational relacorilant as part of two clinical studies: a phase 2 Cushing syndrome study of relacorilant (NCT02804750, EudraCT 2016-000899-23) and a phase 1 oncology study of relacorilant + nab-paclitaxel (NCT02762981). Unique changes to the patients’ tumor characteristics were observed, based on magnetic resonance imaging (MRI) or SSTR imaging. In the Cushing syndrome study, radiologic imaging was included as standard of care, outside the study.

### Case 1: Effect of Relacorilant on Octreotide Scintigraphy in a Patient With Ectopic Cushing Syndrome

A 46-year-old woman with an ectopic ACTH-secreting tumor was enrolled in the phase 2 Cushing syndrome study of relacorilant and received relacorilant 250 mg/day titrated to 400 mg/day. Octreotide scintigraphy (Octreoscan) performed before relacorilant treatment was positive for a lung lesion, but a CT scan and MRI of the lungs did not show any discrete mass at study entry.

**Figure 3:**
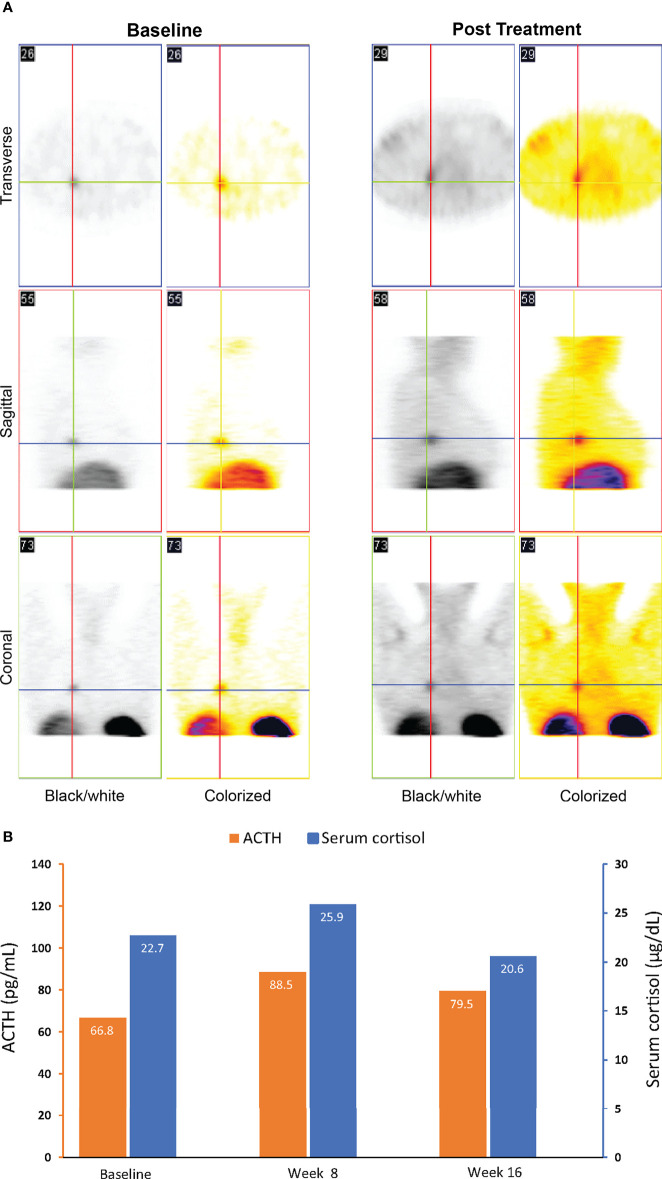
Imaging **(A)** and ACTH and cortisol levels **(B)** for case 1: a 46-year-old woman with an ectopic ACTH-secreting tumor (ectopic Cushing syndrome) treated with relacorilant for 16 weeks. **(A)** Octreotide scintigraphy. Increased uptake on post treatment imaging was consistent with increased expression of SSTR2s following treatment with relacorilant.** (B)** ACTH and cortisol levels before (baseline) and during relacorilant treatment. Normal laboratory ranges: ACTH, 6.0-50 pg/mL; serum cortisol, 4.6-20.6 µg/dL. To convert ACTH values from pg/mL to pmol/L, multiply by 0.22. To convert serum cortisol from µg/dL to nmol/L, multiply by 27.6. ACTH, adrenocorticotropic hormone.

Compared to baseline, repeat octreotide imaging performed after 16 weeks of treatment with relacorilant showed increased uptake at the tumor site (**Figure 3A**). The tumor continued to remain undetectable on repeat CT scans. ACTH and cortisol levels were 66.8 pg/mL (normal range, 6.0–50 pg/mL) and 22.7 µg/dL (normal range, 4.6–20.6 µg/dL), respectively, at baseline. After an initial increase, levels of ACTH and cortisol decreased near or below baseline levels at week 16 (**Figure 3B**), in contrast to the increase seen in patients with ACTH-dependent Cushing syndrome treated with mifepristone (28, 29).

### Case 2: Effect of Relacorilant on SSTR Positron Emission Tomography Imaging in a Patient With an ACTH-Secreting Metastatic Bronchial Carcinoid NET

A 68-year-old man with a metastatic carcinoid NET (bronchial primary) was enrolled in the phase 1 oncology study of relacorilant + nab-paclitaxel. The primary tumor pathology was consistent with a typical carcinoid. Previous treatments included octreotide long-acting release (LAR), everolimus, carboplatin + etoposide, sunitinib, and capecitabine + temozolomide. The patient received relacorilant 200 mg/day on the day before, the day of, and the day after nab-paclitaxel infusion (80 mg/m^2^ administered on days 1, 8, and 15 of a 28-day cycle). During the study, the patient received octreotide LAR 20 mg monthly.

**Figure 4:**
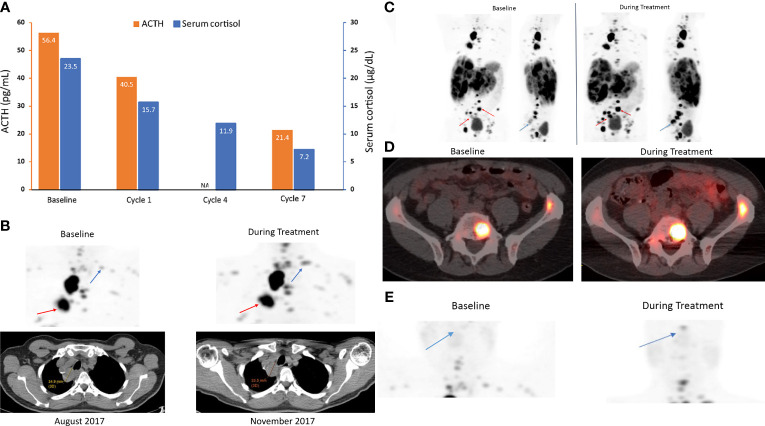
ACTH and serum cortisol levels **(A)** and ^68^Ga-DOTATATE scans **(B–E)** in case 2: a 68-year-old man with a metastatic carcinoid NET treated with 7 cycles of relacorilant + nab-paclitaxel. **(A)** ACTH and cortisol levels before (baseline) and during relacorilant + nab-paclitaxel treatment. Patient received concomitant somatostatin analog. Normal laboratory ranges: ACTH, 6-50 pg/mL; morning serum cortisol, 4.6-20.6 µg/dL. To convert ACTH values from pg/mL to pmol/L, multiply by 0.22. To convert serum cortisol from µg/dL to nmol/L, multiply by 27.6. **(B)** ^68^Ga-DOTATATE scan showed multiple lung and bone lesions at baseline before treatment with relacorilant. Repeat scan during treatment with relacorilant showed increased uptake without change in size of the lesions on CT. **(C)** ^68^Ga DOTATATE scan showed multiple lung, liver, and bone lesions at baseline before treatment with relacorilant. Repeat scan during treatment with relacorilant showed increased uptake. **(D)** ^68^Ga DOTATATE scan of L5 and left iliac bone lesions at baseline. Repeat scan during treatment with relacorilant showed increased uptake. **(E)** Compared with the ^68^Ga-DOTATATE scan before treatment with relacorilant, the repeat scan during relacorilant treatment showed increased uptake at the pituitary gland. ACTH, adrenocorticotropic hormone; NA, not available.

ACTH [56.4 pg/mL [normal range, 6–50 pg/mL)] and cortisol levels [23.5 µg/dL (normal range, 4.6–20.6 µg/dL)] were elevated in this patient at study baseline (**Figure 4A**). ^68^Ga-DOTATATE scans with CT showed increased uptake of the radiotracer at lung and bone lesions during relacorilant treatment compared to baseline without an increase in tumor size (**Figures 4B–D**). ^68^Ga-DOTATATE imaging of the pituitary showed no uptake at baseline (**Figure 4E**). Normally, the pituitary gland expresses SSTR2, and physiologically increased uptake is seen in eucortisolemic patients’ DOTATATE scans (33, 34). During relacorilant treatment, however, the pituitary uptake was restored (**Figure 4E**). ACTH and serum cortisol decreased during concomitant relacorilant and octreotide LAR treatment (**Figure 4A**), suggestive of an increased effect of octreotide LAR due to upregulation of SSTR2 without tumor shrinkage.

### Cases 3 and 4: Effect of Relacorilant on Pituitary Tumor Size in Two Patients With Cushing Disease

**Figure 5:**
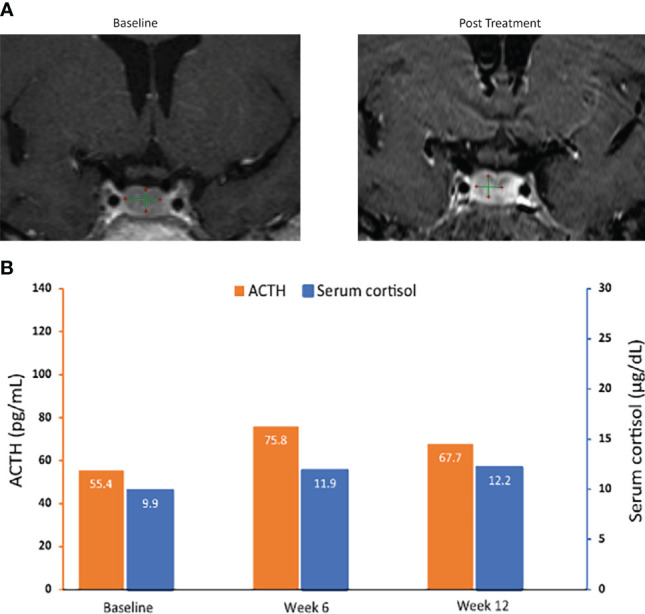
MRI of pituitary macroadenomas **(A)** and ACTH and cortisol levels **(B)** in case 3: a 50-year-old woman with Cushing disease treated with relacorilant for 12 weeks. **(A)** Coronal post contrast T1-weighted MRI obtained at diagnosis (left image) after administration of gadolinium showed a nodular lesion with reduced enhancement in the median and paramedian anterior part of the sellar region compatible with pituitary macroadenoma. It measured 10.01 × 6.29 × 4.91 mm. Pituitary MRI obtained (right image) within 12 weeks after the last dose of relacorilant showed a reduction in the size of the macroadenoma (8.04 × 5.70 × 3.65 mm). **(B)** ACTH and cortisol levels. Normal laboratory ranges: ACTH, 6.0-50 pg/mL; serum cortisol, 4.6-20.6 µg/dL. To convert ACTH values from pg/mL to pmol/L, multiply by 0.22. To convert serum cortisol from µg/dL to nmol/L, multiply by 27.6. ACTH, adrenocorticotropic hormone.

**Figure 6:**
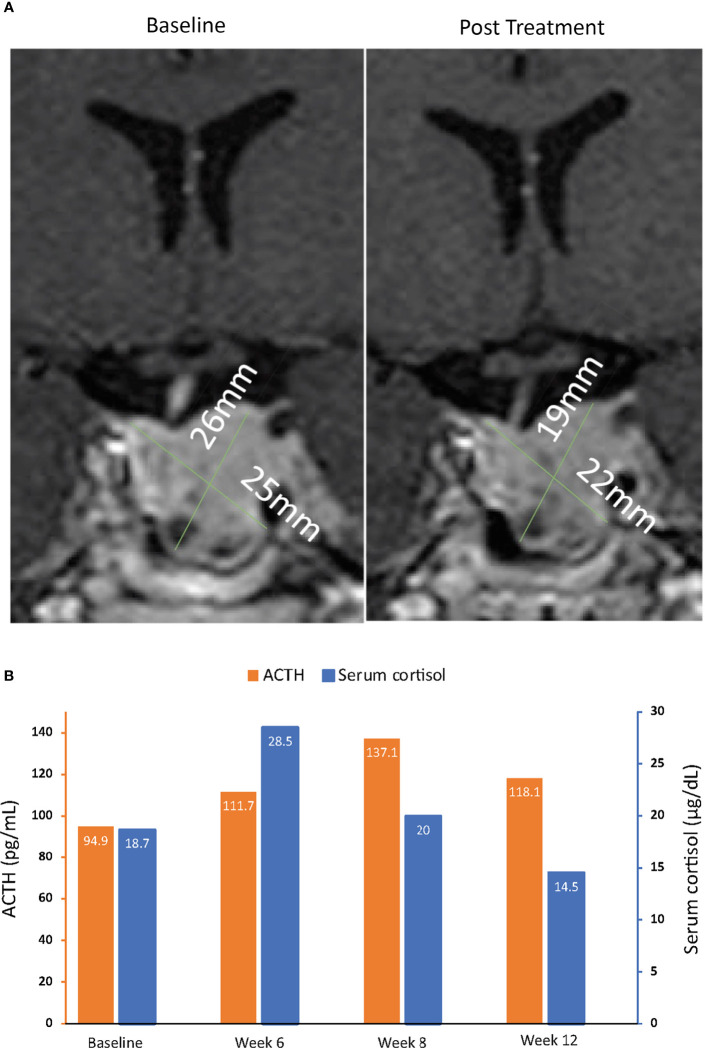
MRI of pituitary macroadenomas **(A)** and ACTH and cortisol levels **(B)** in case 4: a 43-year-old man with Cushing disease treated with relacorilant for 12 weeks. **(A)** Coronal post contrast T1-weighted MRI obtained at diagnosis (left image) after administration of gadolinium showed a pituitary macroadenoma measuring 22 × 25 × 26 mm with suprasellar extension, right displacement of the pituitary stalk, and invasion of the left cavernous sinus. The tumor was isointense to the gray matter and slightly inhomogeneous for the presence of cystic changes in its lower aspect. MRI of the hypophysis obtained within 12 weeks after the last dose of relacorilant (right image), after treatment with relacorilant, showed a reduction in the size of the macroadenoma (21 × 22 × 19 mm). **(B)** ACTH and cortisol levels. Normal laboratory ranges: ACTH, 6.0-50 pg/mL; serum cortisol, 4.6-20.6 µg/dL. To convert ACTH values from pg/mL to pmol/L, multiply by 0.22. To convert serum cortisol from µg/dL to nmol/L, multiply by 27.6. ACTH, adrenocorticotropic hormone.

Two patients with *de novo* Cushing disease due to pituitary macroadenomas received relacorilant 100 mg/day titrated to 200 mg/day as part of a phase 2 Cushing syndrome study prior to previously scheduled transsphenoidal pituitary surgery (35). Patient 3 was a 50-year-old woman with a pituitary macroadenoma measuring 10.01 × 6.29 × 4.91 mm on MRI (tumor volume 155 mm^3^). Patient 4 was a 43-year-old man with a pituitary macroadenoma measuring 22 × 25 × 26 mm (tumor volume 7,150 mm^3^) with suprasellar extension, right displacement of the pituitary stalk, and invasion of the left cavernous sinus on MRI. MRI with gadolinium was conducted before the initiation of relacorilant and within 12 weeks after the last dose of relacorilant. In both patients, imaging revealed reduction in the size of their tumors (**Figures 5A**, **6A**) after treatment with relacorilant. Tumor volume decreased from 155 mm^3^ to 84 mm^3^ for Patient 3 and from 7,150 mm^3^ to 4,389 mm^3^ for Patient 4. Changes in ACTH and serum cortisol levels, as shown in **Figures 5B**, **6B**, showed a similar trend to those of Patient 1 (initial increase followed by reduction below pretreatment levels).

## Discussion

SSTR2s are expressed in a variety of tumor types, which has led to the use of SSTR2-targeting analogs for diagnosis and treatment. However, the effects of GR modulation on SSTR2 have not been well studied. The preclinical data as well as several clinical case observations reported here illustrate the potential effects of relacorilant on ACTH-secreting NETs. In the *in vitro* analysis, selective GR modulation with relacorilant inhibited glucocorticoid-mediated suppression of SSTR2 in the murine At-T20 cell line. Imaging and laboratory data from four patients with ACTH-secreting NETs showed increased uptake of radiotracer *via* SSTR2-based imaging and a reduction in pituitary corticotroph tumor size following treatment with relacorilant.

Glucocorticoids induce downregulation of SSTR2, which can explain the low SSTR2 expression reported in tumors derived from patients with Cushing disease (20). In the At-T20 cell line, dexamethasone-mediated suppression of SSTR2 mRNA was reversed by selective GR modulation with relacorilant. At higher concentrations of relacorilant, SSTR2 mRNA expression even increased above basal levels in the At-T20 cell line. Studies of the regulatory effects of somatostatin in normal rat pituitary cells and in healthy humans have shown that treatment with somatostatin does not inhibit basal or CRH-stimulated ACTH secretion (1, 16, 36). However, in patients with Nelson’s syndrome and elevated plasma ACTH following bilateral adrenalectomy for Cushing disease, somatostatin infusion was shown to decrease ACTH secretion (37). In a separate analysis of patients with primary adrenal insufficiency, somatostatin injection also resulted in a reduction in ACTH (38). Patients in both studies had been receiving glucocorticoid replacement therapy, which was withheld prior to the administration of somatostatin. Together with the findings of the current study, these data suggest that either a lack or an excess of glucocorticoids may lead to abnormal SSTR2 expression (upregulation in adrenal insufficiency and downregulation in Cushing syndrome). GR modulation with relacorilant may overcome the inhibitory effect of glucocorticoids on SSTR2 expression, restoring the efficacy of the endogenous somatostatin and exogenous somatostatin analogs.

The two patients with ectopic tumors showed increased uptake of the radioactive somatostatin analog used for imaging NETs with relacorilant administration. This result is notable, as in up to 27% of patients with ectopic Cushing syndrome, the tumor source is not localized even after long-term follow-up (15). While increased uptake could also have occurred because of interval increases in the size of the lesions, there was no evidence of a change in tumor size in these patient cases based on CT imaging. SSTR2 is normally expressed in the pituitary gland (33, 34), and increased uptake on ^68^Ga-DOTATATE scan is seen in eucortisolemic patients. In the patient with an ACTH-secreting metastatic bronchial NET, there was no physiologic uptake of ^68^Ga-labeled somatostatin analog in the pituitary gland at baseline. Of note, ectopic ACTH secretion is common in lung carcinoid tumors but is not always associated with overt cortisol excess (39). Because of the low differentiation of these tumors, the ACTH secreted by the tumors in most cases is biologically inactive (ACTH-like peptides, also referred to as ACTH precursors) but can cross-react with commercially available ACTH assays. The lack of ^68^Ga-labeled somatostatin analog uptake in the pituitary gland, along with the elevated serum cortisol and ACTH levels at baseline, suggests that this patient had some degree of cortisol excess at baseline; however, no formal evaluation for Cushing syndrome (eg, urinary free cortisol, dexamethasone suppression testing, or late-night salivary cortisol) was required for enrollment in the oncology study. In this patient, treatment with relacorilant reversed the effect of cortisol on the SSTR2s in the pituitary, resulting in restoration of SSTR2 expression in the pituitary and visualization in the repeat scans. This patient was also receiving concomitant nab-paclitaxel, but the authors are not aware of any studies suggesting that nab-paclitaxel has an effect on SSTR2. These cases highlight the potential effects of GR modulation with relacorilant in instances of ectopic ACTH secretion and suggest that relacorilant can enhance SSTR-based imaging, which may improve diagnostic accuracy.

The increased expression of SSTR2 with relacorilant was also supported by changes in the patients’ ACTH and cortisol levels. In patients 1, 3 and 4, ACTH and cortisol levels initially increased and then decreased later during treatment. The initial increase was expected based on experience with the GR antagonist mifepristone (28). The mechanisms for the eventual decrease in ACTH and cortisol levels with relacorilant are not yet fully understood. While the decrease in ACTH and cortisol during relacorilant treatment might reflect an exhaustion of the stimulatory effect of relacorilant, this effect has not been observed with mifepristone. In patients with Cushing disease, mifepristone use is associated with dose-dependent increases in ACTH and cortisol, and ACTH levels generally remain elevated over time with continued treatment (29). Furthermore, mifepristone pretreatment in patients with Cushing disease was not shown to affect ACTH and cortisol levels in response to acute octreotide administration (40). The overexpression of SSTR2 observed at higher doses of relacorilant in the *in vitro* analysis offers another possible explanation for the effects observed with relacorilant. In the previous studies of mifepristone in NET cell lines, mifepristone reversed the effects of dexamethasone but was not associated with SSTR2 overexpression (27). Together, these findings suggest a potential difference in effect between mifepristone and relacorilant, in which relacorilant-induced increased SSTR2 expression on the tumor can increase the efficacy of endogenous and exogenous somatostatin on ACTH secretion and tumor proliferation. In Patient 2, who received octreotide LAR along with relacorilant, the levels of ACTH and cortisol decreased throughout relacorilant treatment, without the initial increase that was observed in patients not receiving concurrent somatostatin analogs. A possible explanation is that in the presence of high somatostatin levels achieved with exogenous administered somatostatin analogs, even lower doses of relacorilant may lead to sufficient upregulation of SSTR2 to enhance the effect of somatostatin analogs on the secretory function of the NET; however, this would need to be confirmed by further research in a larger, more homogenous population.

Somatostatin analogs have been shown to inhibit tumor hypersecretion of peptides and slow tumor growth in gastrointestinal cancers, including NETs (9–12), and expression of SSTR2 has been associated with improved survival in patients with gastropancreatic NETs (41). The antisecretory and anti-proliferative effects of somatostatin and its analogs are mediated by both direct and indirect mechanisms (3, 42). Direct effects include cell cycle arrest, inhibition of growth factor signaling, and apoptosis through the regulation of MAP kinase and phosphotyrosine phosphatase activities upon activation of SSTR2. Indirect effects include inhibition of tumor angiogenesis, secretion of tumor-promoting signals from immune cells, and secretion of growth factor. Octreotide has also been shown to reduce tumor volume in patients with growth hormone-secreting and thyroid-stimulating hormone-secreting pituitary tumors (3, 42). In the clinical case examples of the current study, we observed a decrease in tumor size in two patients with *de novo* Cushing disease due to macroadenomas (patients 3 and 4) following treatment with relacorilant. While spontaneous tumor regression cannot be ruled out (although extremely rare) (43), the changes in ACTH and cortisol levels that occurred in these patients during relacorilant treatment, characterized by early increases followed by reductions later during treatment, are consistent with the hypothesized inhibition of ACTH by endogenous somatostatin due to upregulation of SSTR2, as also seen in Patient 1. Together, these data suggest that relacorilant-mediated upregulation of SSTR2 provides more targets for somatostatin and somatostatin analogs, which can lead to tumor shrinkage in ACTH-secreting pituitary tumors.

There are a number of limitations to the data presented in this report. Although the murine corticotroph tumor At-T20 cell line is the most frequently studied model for Cushing disease, the *in vitro* findings may not necessarily translate to human cells. Thus, one cannot rule out the possibility of another mechanism for the trends in ACTH and cortisol levels observed in the patient cases. The small number and heterogeneous nature of the clinical cases, including concomitant therapies, must also be considered when interpreting the clinical observations. The lack of SSTR2 imaging or immunohistochemical analysis of SSTR2 expression before and after relacorilant treatment is another limitation for patient cases 3 and 4; however, SSTR2 imaging is not part of the standard diagnostic evaluation of ACTH-secreting pituitary tumors (44).

Based on these findings, additional examination should be carried out to formally assess and elucidate the tumor-specific effects of relacorilant in patients with ACTH-producing NETs to determine whether it has a potential diagnostic role and antitumor effects. A therapeutic trial that could sensitize ACTH-secreting pituitary tumors to endogenous somatostatin prior to surgery could be beneficial, particularly in patients with invasive macroadenomas. Ongoing preclinical studies in human pituitary cell lines and the phase 3 study of relacorilant in patients with Cushing syndrome (clinicaltrials.gov NCT03697109), which includes tumor imaging, may provide additional insight.

## *In Vitro* Methods

### Cell Culture

At-T20 mouse pituitary tumor cells were obtained from ATCC (CCL-89) and cultured in high-glucose Dulbecco’s minimal essential medium (DMEM) complete media [10% fetal calf serum + penicillin and streptomycin (Penn/Strep)]. For compound treatment, 96-well plates were seeded with 50,000 cells and allowed to adhere for 6 h in complete media containing charcoal-stripped serum. Agonist treatment with a dexamethasone concentration gradient was carried out for 24 h [0.2% final dimethylsulfoxide (DMSO)]. For antagonist assays, the cells were pre-treated for 30 min with a relacorilant concentration gradient prior to the addition of 100 nM (EC_max_) dexamethasone and incubated for 24 h (0.2% final DMSO). After treatment, the medium was removed, and cells were lysed directly in lysis buffer (buffer RLT Qiagen RNeasy) followed by total RNA extraction.

### RNA Isolation

Total RNA from At-T20 cellular lysates was isolated using Qiagen RNeasy Mini kit (Qiagen 74104) by following the manufacturer’s recommended instructions. RNA was eluted in a 100 µL volume of nuclease-free elution buffer and stored at -20°C until use. Contaminating genomic DNA was eliminated by the inclusion of a deoxyribonuclease treatment step (deoxyribonuclease I at 8 U per 100 µL of eluate) (45). Total RNA yield and purity were measured by spectrophotometric analysis (A_260_ to A_280_ ratio) using a Nanodrop 1000 instrument.

### Reverse Transcription and Real-Time Quantitative PCR

Reverse transcription and real-time qPCR were performed as previously described (45). Total RNA (0.2–1.0 µg) was reverse transcribed in 20 µL reaction volume using a high-capacity cDNA reverse transcription kit (Applied Biosystems; 4368814) with random hexamers. Real-time qPCR experiments were performed in a 96-well plate using an Applied Biosystems StepOnePlus real-time PCR instrument. For each sample, the expression of SSTR2 was compared with the expression of glyceraldehyde 3-phosphate dehydrogenase (GAPDH) mRNA, with the latter included as a housekeeping gene for endogenous control. Taq-Man gene expression assays were obtained from Life Technologies and consisted of a 20X mix of unlabeled PCR primers for mouse SSTR2A (Life Technologies; Mm03015782_s1) and mouse SSTR2B (Life Technologies; Mm00436685_g1) and for mouse GAPDH (Life Technologies; Mm99999915_g1) and TaqMan MGB probe (FAM dye labelled). The reaction mixture for real-time qPCR contained 9.0 µL cDNA solutions (20–100 ng). Each of the two primers and the MGB probe were used at 0.9 µM and 0.25 µM, respectively, and 1X TaqMan Universal Master Mix II with UNG (Applied Biosystems; 4440038). The mixture was activated (2 min, 50°C), denatured (10 min, 95°C) and subjected to 40 amplification cycles (15 sec, 95°C; 1 min, 60°C) with a single measurement of fluorescence for both SSTR and GAPDH primer sets.

### Data Analysis

TaqMan qPCR data were analyzed using StepOnePlus software version 2.3. Amplification plots were visualized across the entire 96-well plate for SSTR2A/B probe sets and GAPDH. Fractional cycle (C_T_) values were returned by manually setting the threshold to intersect at the linear phase of amplification plots (defined manually at 0.1) (45). No-treatment control sample was selected as the calibrator, and the data were analyzed relative to the calibrator. The comparative ΔΔC_T_ method was used for data analyses.

## Data Availability Statement

The original contributions presented in the study are included in the article/supplementary materials. Further inquiries can be directed to the corresponding author.

## Ethics Statement

The studies involving human participants were reviewed and approved by the institutional review board at each study center. The patients/participants provided their written informed consent to participate in this study.

## Author Contributions

Study concept and design: AM. Study investigators who provided study materials and/or patients: RP, PM, MT, RF, SP, and CS. Analyzed and interpreted preclinical data: UB and AM. Analyzed and interpreted clinical data: RP, PM, MT, RF, SP, CS, and AM. Wrote manuscript or critically revised it for content: All authors. Reviewed final manuscript and gave approval for submission: All authors.

## Funding

The studies were funded by Corcept Therapeutics. Open Access publication fees were paid by Corcept Therapeutics.

## Conflict of Interest

RP: Consultant: Ferring, Ipsen, Novartis, Pfizer, ViroPharma-Shire; Speaker: Novartis, ViroPharma-Shire; Research support: Corcept Therapeutics, Novartis, ViroPharma-Shire; Grant support: IBSA, Novartis, Pfizer, ViroPharma-Shire. PM: Consultant: Atlas, Alessa, EpiAxis, Rascal and AstraZeneca. MT: Consultant: HRA Pharma; Research support: Corcept Therapeutics. CS: Consultant: Ipsen, ViroPharma-Shire. UB: Consultant: Corcept Therapeutics; Employee: Sygnature Discovery, Ltd. AM: Employee: Corcept Therapeutics.

The authors declare that the studies received funding from Corcept Therapeutics (Menlo Park, CA, USA). The funder had a role in study design, data collection and analysis. MA was employed by the company Corcept Therapeutics and, as an author of the manuscript and employee of Corcept Therapeutics, had a role in the study design, the decision to publish, the interpretation of clinical data, the revision of the manuscript, and approval of the final manuscript to submit. UB was employed by the company Sygnature Discovery Ltd. and, as an author of the manuscript and employee of Sygnature Discovery Ltd., supported by the funder, had a role in the preclinical data analysis and interpretation, decision to publish, revision of the manuscript, and approval of the final manuscript to submit. Open Access publication fees were paid by Corcept Therapeutics.

## Publisher’s Note

All claims expressed in this article are solely those of the authors and do not necessarily represent those of their affiliated organizations, or those of the publisher, the editors and the reviewers. Any product that may be evaluated in this article, or claim that may be made by its manufacturer, is not guaranteed or endorsed by the publisher.

## Abbreviations

ACTH, adrenocorticotropic hormone; CRH, corticotropin-releasing hormone; CT, computed tomography; DMEM, Dulbecco’s minimal essential medium; DMSO, dimethylsulfoxide; GAPDH, glyceraldehyde 3-phosphate dehydrogenase; GR, glucocorticoid receptor; LAR, long-acting release; MRI, magnetic resonance imaging; NET, neuroendocrine tumor; PET, positron emission tomography; qPCR, quantitative PCR; SD, standard deviation; SGRM, selective glucocorticoid receptor modulator; SSTR, somatostatin receptor; SSTR1-5, somatostatin receptor subtypes 1-5.
